# Synergistic Effect of Halloysite Nanotubes and Glycerol on the Physical Properties of Fish Gelatin Films

**DOI:** 10.3390/polym10111258

**Published:** 2018-11-13

**Authors:** Xiaohu Qiang, Songyi Zhou, Zhuo Zhang, Qiling Quan, Dajian Huang

**Affiliations:** School of Materials Science and Engineering, Lanzhou Jiaotong University, Lanzhou 730070, China; zsy1164826030@126.com (S.Z.); zhang1466rose@163.com (Z.Z.); quanql0329@163.com (Q.Q.); huangdj2015@yeah.net (D.H.)

**Keywords:** fish gelatin, halloysite nanotubes, glycerol, mechanical properties, water resistance

## Abstract

Fish gelatin (FG)/glycerol (GE)/halloysite (HT) composite films were prepared by casting method. The morphology of the composite films was observed by scanning electron microscopy (SEM). The effects of HT and GE addition on the mechanical properties, water resistance and optical properties of the composites were investigated. Results showed that with increasing GE content, the elongation at composite breaks increased significantly, but their tensile strength (TS) and water resistance decreased. SEM results showed that GE can partly promote HT dispersion in composites. TS and water resistance also increased with the addition of HTs. Well-dispersed HTs in the FG matrix decreased the moisture uptake and water solubility of the composites. All films showed a transparency higher than 80% across the visible light region (400–800 nm), thereby indicating that light transmittance of the resulting nanocomposites was slightly affected by GE and HTs.

## 1. Introduction

Recently, studies using natural polymer materials to replace synthesis plastic for preparing packaging films have attracted wide attention because of the pollution problems caused by synthetic plastic [1,2,3,4]. When natural polymer materials are mentioned, gelatin, with low cost, biocompatibility, and biodegradability, is one of the most promising [5,6,7,8]. Fish gelatin (FG), a type of gelatin that can provide materials with ultraviolet and oxygen barrier properties, can be extracted from fish bones and skin, and has been used an alternative raw material for a wide range of applications [9,10,11]. However, FG’s inflexible and hydrophilic character, and consequently its poor mechanical properties in the presence of humid environments, limit its application [10,12,13]. As a consequence, the flexible properties and water-resistance of FG-based films should be improved further to meet the demand of practical applications.

An effective strategy exploring how to improve the properties of gelatin-based films is adding plasticizer into the gelatin matrix [14,15,16]. Plasticizers could break the polymer–polymer interactions (such as hydrogen bonds and Van der Waals forces) and form secondary bonds to gelatin molecular chains, thereby increase the flexibility of the films and preventing them from cracking during packing and transportation [16,17,18]. Among possibilities of additives that can be used as plasticizer in gelatin-based films, glycerol (GE) is the most commonly used in gelatin-based films [18,19,20]. Compared with other plasticizers such as sorbitol, polyethylene glycol and diethylene glycol, GE has smaller molecular dimensions and higher hydroxyl density, so it is widely used as a plasticizer into a hydrophilic biopolymers matrix.

Incorporating inorganic nanoparticles such as clay, graphene, zinc oxide and carbon nanotubes into the gelatin matrix has been shown to be an effective method to improve its thermal, mechanical, and barrier properties. Farahnaky et al. found that the mechanical properties and water-resistance of gelatin films were improved by the addition of montmorillonite [21]. Shankar et al. prepared gelatin/ZnO nanoparticle nanocomposite films using various types of ZnO nanoparticle [22]. The results showed that the moisture content, water contact angle, water vapor permeability, and elongation at breaks of ZnO nanoparticle incorporated films increased, while tensile strength and modulus of elasticity decreased compared with the control gelatin film. Li et al. studied the effect of the concentration of laponite on the physicochemical properties of a gelatin/laponite film [23]. They found that gel strength, mechanical properties, water vapor permeability and water solubility of gelatin/laponite composite films were greatly enhanced with an increase of the concentration of laponite.

Halloysite nanotubes (HTs) are natural aluminosilicates from a clay mineral with a hollow nanotubular structure [24,25]. HTs have been developed to use in controlled release [26], templates [27], and sorption [28] during the past decades. Recently, HTs have been successfully developed as a reinforcing filler to improve the properties of biopolymers. For instance, the mechanical properties and thermal stability of starch films were improved by the addition of HTs [29,30]. Soheilmoghaddam et al. found that the tensile strength of regenerated cellulose film increased from 35.30 to 60.50 MPa when 8 wt % HTs were added, without loss of ductility [30]. Liu et al. have prepared chitosan/HTs composites via solution casting and the FE-SEM results indicated that HTs are uniformly dispersed in the chitosan matrix [31]. The tensile strength of chitosan was improved by HTs. The low cost and environmentally friendly nature make HTs competitive with other reinforcing fillers for biopolymers. In addition, unlike two-dimensional platelet-like nanoclays [32,33], the rod-like geometry of HTs hardly intertwines, which makes HTs disperse better in a polymer matrix [34].

The objective of this work is to investigate the combined effect of GE and HTs on the properties of FG-based composites. For this purpose, we prepared a series of films containing different amounts of HTs and GE and characterized them using field emission scanning electron microscopy (FE-SEM) and Fourier Transform Infrared Spectroscopy (FT-IR). The mechanical properties, water-resistance, and optical properties of composite films were investigated to establish a structure–property correlation between HTs, GE and the FG matrix.

## 2. Materials and Methods

### 2.1. Materials

FG was purchased from Taosheng Chemical Co., Ltd. (Guangzhou, China). HTs were purchased from Yuanxin Nanotechnology Co., Ltd. (Guangzhou, China). GE is analytical grade, and used without further purification.

#### Preparation of Composite Films

Preparation of composite films is on the basis of the method in the literature. FG was added into distilled water at 50 °C with magnetic stirring for 1 h to prepare the FG solution (4%, *w*/*w*). Then, the GE, as a plasticizer, was added into the FG solution at a certain loading (0, 20, 30, 40% (*w*/*w*)) with respect to FG weight. The obtained mixture was stirred for 1 h to acquire homogeneous solutions. HTs dispersions (0.5%, *w*/*w*) were prepared by adding appropriate amounts of HTs into distilled water under mechanical agitation at 500 rpm for 1 h at room temperature. The obtained HTs dispersions were added in the four types of FG/GE composites (pure FG, FG/GE (20%), FG/GE (30%), and FG/GE (40%)) at 0%, 4% and 8% (*w*/*w*) loadings with respect to FG weight, respectively. Following this, the obtained mixtures were stirred for 1 h at 50 °C to acquire homogeneous solutions. These film-forming solutions were placed at room temperature for 1 h to remove the bubble. After that, the mixtures were poured into 9 cm diameter polycarbonate petri dishes and evaporated into the atmosphere for 72 h; the resulting films were dried in a vacuum oven (at 70 °C) for 6 h to remove traces of the solvents. The named rule of composite films in this work is directly named by the mass fraction of GE and HTs: for example, composite films with 40 wt % GE loading are named FG/GE40, and composite films with 40 wt % GE and 10 wt % HTs are named FG/GE40/HTs10.

### 2.2. Characterization

The morphology of samples was observed by a field emission scanning electron microscope (FESEM, JEOL JSM-6701F, Japan Electron Optics Limited, Tokyo, Japan), after coating the sample with a layer of gold. Water contact angles (WCA) were measured with a Contact Angle System OCA 20 (Dataphysics, Stuttgart, Germany) at ambient temperature by injecting 5 μL of water droplets onto the surface of films. Average WCA values were determined by measuring the same sample at five different positions. Transmission electron microscopy (TEM) analysis of samples was carried out with a TECNAIG2-F30, FEI.

X-ray diffraction (XRD) patterns were collected using an XRD-7000 (XRD, Shimadzu XRD-7000, Shimadzu Co., Ltd., Kyoto, Japan) diffractometer with Cu Ka radiation, scanning from 3° to 40° at 3°/min. Attenuated total reflectance Fourier transform infrared spectra (ATR-FTIR) were recorded on an FTIR Spectrophotometer (Thermo Nicolet, iS10, Thermo Fisher, Waltham, MA, USA) in the range of 4000–400 cm^−1^. Film thickness was studied to the nearest 0.001 mm with a digital micrometer (Micrometer, Wuxi, China) at 9 random positions around the film.

### 2.3. Tensile Tests

Tensile tests were performed using a an AG-IS material testing machine (Shimadzu Co., Ltd., Kyoto, Japan) equipped with a 200 N load cell at room temperature, with a gauge length of 40 mm and crosshead speed of 10 mm/min. The results were evaluated as an average of at least 5 measurements.

### 2.4. Moisture Uptake

Moisture uptake of the films was measured on samples cut into small pieces (2 cm × 3 cm). Samples were first dried in an oven at 70 °C for 8 h. After weighing, the samples were stored over NaCl saturated salt (76% RH) for 72 h to ensure equilibrium of the moisture before reweighing. The moisture uptake of the samples was calculated as follows:

(1) Moisture uptake = [*W*_1_ − *W*_0_]/*W*_0_ × 100%

where *W*_0_ and *W*_1_ are the weight of the samples before exposure to 76% RH and at equilibrium, respectively. All tests were carried out with three replicates and an average value for each sample was obtained.

### 2.5. Water Solubility

Water solubility of the films was studied by the following method: Six pieces (2 cm × 3 cm) of film dried in an air-circulating oven at 70 °C until reaching constant weight and were weighed (*M*_0_). After this, the films were immersed in 50 mL of distilled water. After 24 h of storage in an environmental chamber at 25 °C, samples were recovered and gently rinsed with distilled water. After drying in an air-circulating oven at 70 °C until reaching constant weight, the films were weighed again (*M*_1_).

The water solubility of films was calculated by the following equation:

(2) Water solubility = [*M*_0_ − *M*_1_]/*M*_0_ × 100%

### 2.6. Optical Properties

The opacity of composite films was studied by a UV–visible spectrophotometer (N4, INESA (Group) Co., Ltd., Shanghai, China). Composite films were cut into rectangular strips and placed on the two outer sides of a spectrophotometer cell and light transmission was recorded at room temperature in steps of 1 nm, in the range 200–800 nm.

### 2.7. Statistical Analysis

Averages and standard deviations from at least three measurements of each sample were reported in this study. Student’s t-test was used for analysis of test results at the significance level of *p*-value < 0.05.

## 3. Results

### 3.1. Film Thickness

**Figure 1:**
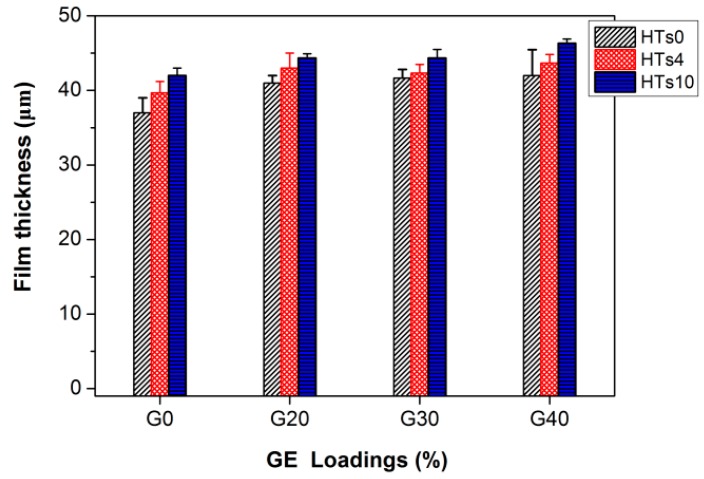
Film thickness of fish gelatin (FG)/glycerol (GE)/halloysite (HT) composites with various amounts of HT and GE loadings.

Film thickness is an important parameter in the calculation of mechanical and other physical properties. As shown in Figure 1, the addition of HT and/or GE generated an increase in the thickness of the composites films. The film thickness increased from 37.0 μm for the control FG film to 46.3 μm for the FG/GE40/HTs10 composite film. This phenomenon could be due to the increase in dry matter content of composites.

### 3.2. SEM Analysis

**Figure 2:**
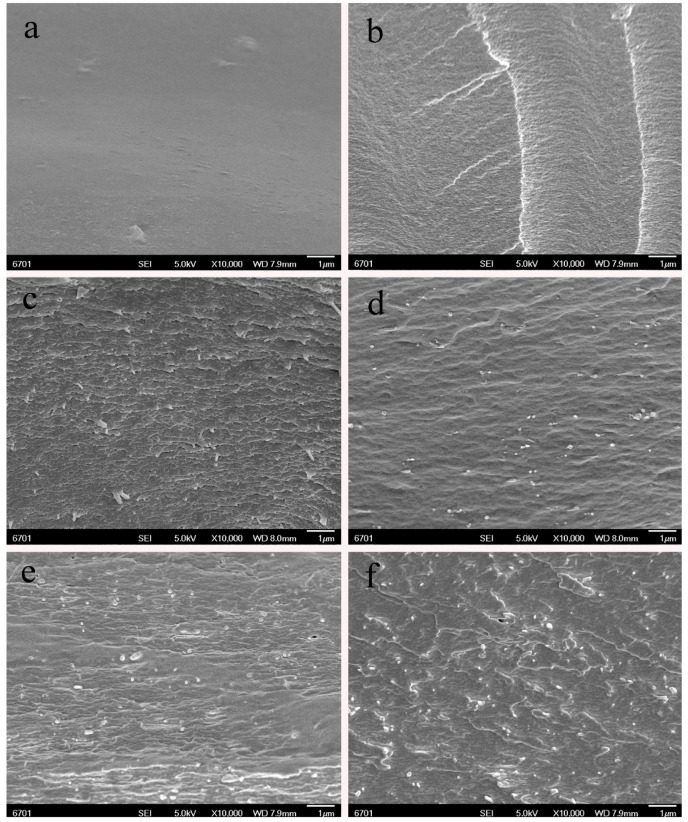
SEM micrographs of (**a**) pure FG, (**b**) FG/GE40, (**c**) FG/HT4, (**d**) FG/GE40/HTs4, (**e**) FG/HTs10 and (**f**) FG/GE40/HTs10.

The degree of filler dispersion is a key factor for the final properties of the nanocomposite [35]. SEM micrographs of the fracture surface of selected films are presented in Figure 2. The SEM micrographs of pure FG and FG/GE40 films are shown in Figure 2a,b, respectively. It can be observed that fracture surfaces of the two types of film are smooth and homogeneous, without pores or cracks. Compared with the pure FG film, the fracture surface of FG/GE40 film shows a more dense structure, indicating that there is good compatibility between FG and GE, and that the GE molecular can insert into the FG molecular chains.

**Figure 3:**
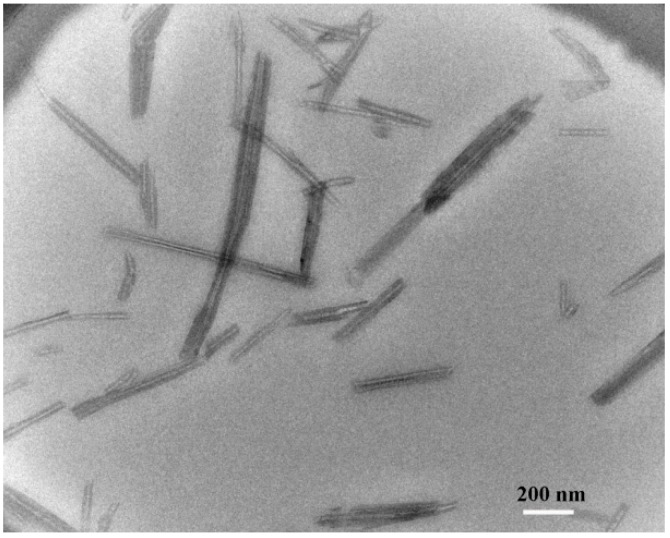
TEM micrographs of FG/GE40/HTs4.

The SEM micrographs of FG/HTs4 and FG/GE40/HTs4 films are shown in Figure 2c,d, respectively. These two films showed rougher fractured surface compared with the films without HTs. The bright dots shown in Figure 2c,d are terminals of HTs, which demonstrates that HTs were embedded in the FG matrix successfully. A good dispersion of HTs in the FG or FG/GE40 matrix was observed without obvious agglomeration in the two types of composites, indicating that the HTs, GE and FG were compatible. In order to further observe the dispersion of HTs in the polymers, TEM was used to investigate the FG/GE40/HTs4 sample and the obtained micrographs are displayed in Figure 3. As shown in Figure 3, the HTs possess a hollow cylindrical shape and show a transparent central area along the cylinder with an open end. The HTs were well dispersed in the FG/GE matrix. The well-dispersed HTs in the FG or FG/GE40 matrix can be attributed to the following reasons: First, the nanotube morphological character of HTs and the relatively weak nanotube—nanotube interactions allowed to disperse easily in FG by shear force. Second, HTs can interact with FG through hydrogen bonding interactions, which also led to its effective dispersion. As the content of HTs was increased to 10%, some agglomerations were found in the two types of composites (Figure 2e,f), which may be mainly due to the Van der Waals force among HTs. It can be seen that the HTs in the FG/HTs composites exhibit more agglomeration than that of FG/GE40/HTs composites. This phenomenon may be ascribed to the interaction of hydrogen bonds between GE and HTs, and GE could promote the dispersion of HTs in the composites. A similar phenomenon was reported by Lavorgna et al., when they found that the presence of GE improved the chitosan intercalation in the silicate galleries of montmorillonite and prevented the flocculation process, leaving the MMT stacks randomly orientated in the composites [36].

### 3.3. XRD Analysis

**Figure 4:**
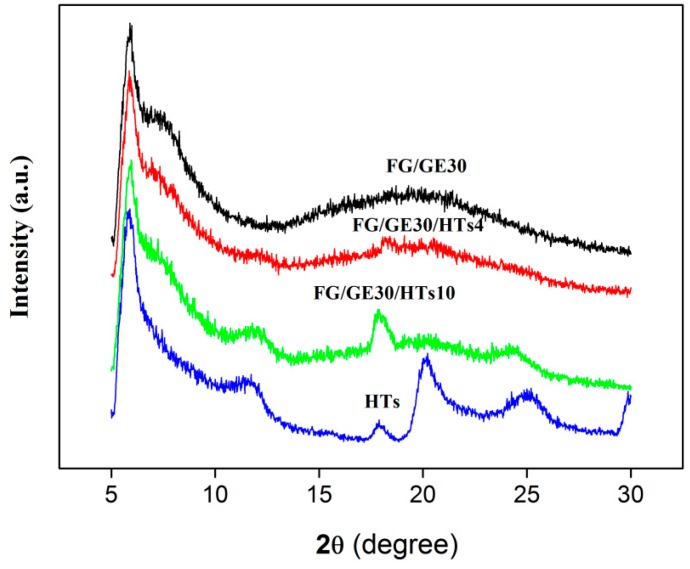
XRD patterns of HTs and FG/GE30 at 0, 4 and 10 wt %.

Figure 4 shows the XRD patterns of HTs and FG/GE30 composites with HT loadings of 0%, 4% and 10%. The basal spacing (001) of pristine HTs was approximately 0.72 nm, corresponding to 2θ = 12.0° [37], with other peaks appearing at higher angles of 2θ = 20.22° and 2θ = 24.52°; these are assigned to (020), (110) and (002), respectively [31]. Typical XRD peaks for HNT powders appear at 2θ = 18°, in accordance with reflection planes (002) of gibbsite, which is an impurity in the HTs. Owing to its strong orientation, the peak of gibbsite at 2θ = 18° appeared obviously even at a small loading. This XRD peak of gibbsite as an impurity in the HTs also was found in other studies. The FG/GE30 film showed a broad peak in the 2θ range of 6.2°–9.5°, which is a characteristic of amorphous proteins [38]. As for the FG/GE30/HTs composite, the intercalation of FG or GE molecules into the interlayers of HTs’ hollow tubular structure does not take place, as the d-spacing values for their weak (001) peaks remain almost unchanged [33]. This peak gradually increased with increasing HT content of the FG/GE30 matrix. In addition, the peaks at 20.22° and 24.52° become invisible. This finding is in good agreement with previous work based on polymer/HTs composites [31].

### 3.4. FTIR Analysis

**Figure 5:**
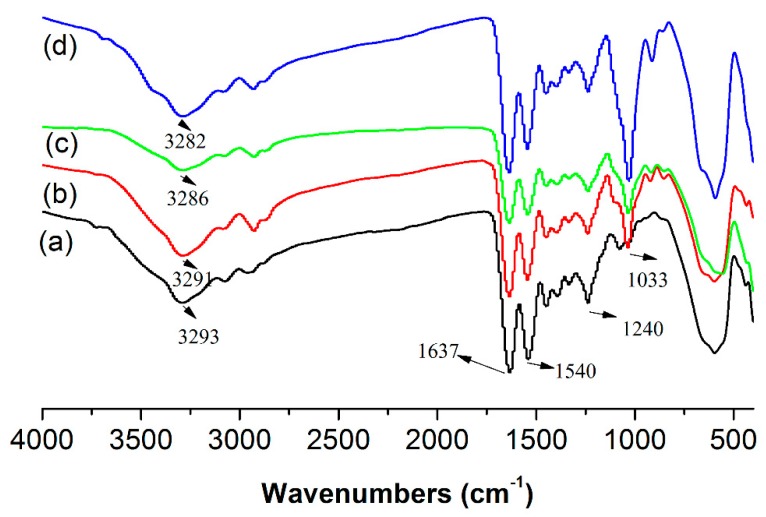
ATR-FTIR spectra of (**a**) pure FG, (**b**) FG/GE30, (**c**) FG/GE30/HTs4 and (**d**) FG/GE30/HTs10.

**Scheme 1:**
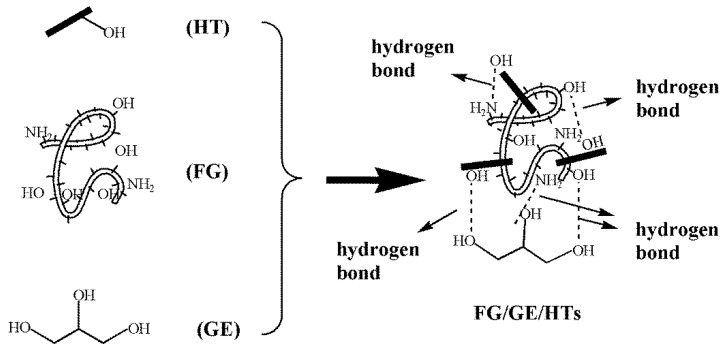
Schematic illustration of the mechanism of HTs in the FG/GE composites.

ATR–FTIR analysis was performed to study the interactions between HTs and the FG matrix, and the results are presented in Figure 5. FG is an amphoteric polyelectrolyte with both positively and negatively charged amino acids dispersed on the chain backbone [21]. The FT-IR spectrum of pure FG film showed the characteristic amide I, amide II and amide III bands. As shown in Figure 1a, the pure FG film showed a major band at 1637 cm^−1^ (amide I band), which is related to the C=O stretching/H bonding in combination with COO [21]. The peak at 1540 cm^−1^ was because of amide-II, produced by bending vibrations of N–H groups and stretching vibrations of C–N groups [21]. The peak at 1240 cm^−1^ can be attributed to the vibration of C–N and N–H groups of bound amide, or vibration of CH_2_ groups of glycine [39]. The peak at 1033 cm^−1^ is related to the interaction between GE (OH group of GE) and FG [40]. In the spectrum of FG/GE30 (Figure 1b), the stretching and bending vibrations of the hydrogen bonding –OH group occurred at 3291 cm^−1^ [20]. The peak (3291 cm^−1^) is observed to shift to 3286 and 3282 cm^−1^ (Figure 1c,d) after incorporation of 4% and 10% HTs into the FG/GE30 matrix, respectively. These behaviors indicate that HT can interact with FG and GE, and partially destruct the hydrogen bonding between FG and GE [20]. The mechanism of interactions among FG, GE and HTs is illustrated in Scheme 1. A quite similar tendency was found for attapulgite introduction into the PVA/chitosan matrix [31]

### 3.5. Mechanical Properties

**Figure 6:**
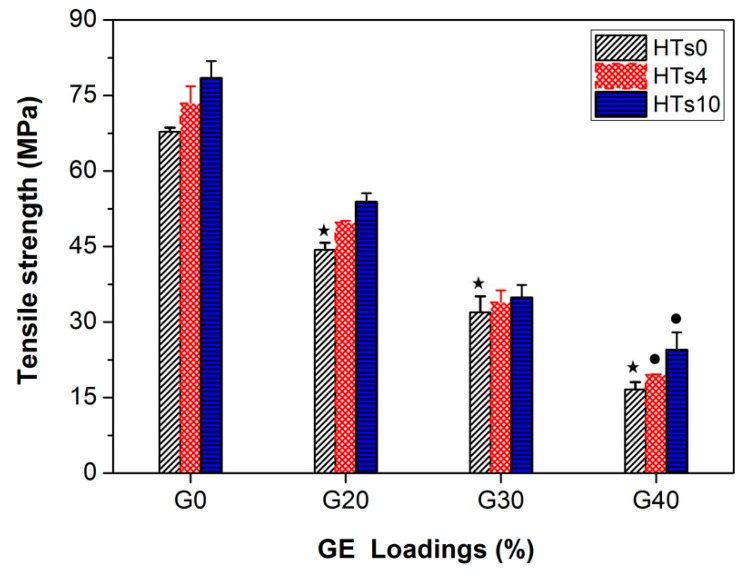
Tensile strength (TS) of FG/GE/HT composites with various HT and GE loadings. * denotes significant difference between FG films containing different percentages of GE (*p* < 0.05). ● denotes significant difference in FG/GE40 films containing different percentages of HT compared with FG/GE40 only film (*p* < 0.05).

**Figure 7:**
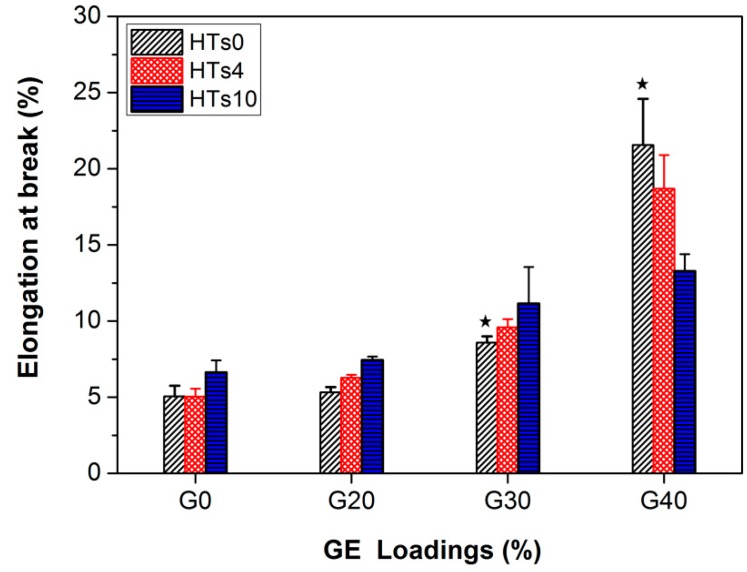
Elongation at break (EB) of FG/GE/HT composites with various HT and GE loadings. * denotes significant difference between FG films containing different percentages of GE (*p* < 0.05).

Mechanical properties are one of the most important parameters to packaging materials. Mechanical properties of composite films were characterized by tensile strength (TS) and elongation at break (EB) [41]. The results of TS are shown in Figure 6. As expected, the TS of films decreased prominently from 65 to 15 MPa by elevating the proportion of GE from 0% to 40% *w*/*w* (*p* < 0.05). The effect of GE addition on the EB of films has an inverse behavior (Figure 7) compared with their correspondent TS. Meanwhile, the increase of GE addition from 0% to 40% caused EB to greatly increase (*p* < 0.05). This may be due to the role of GE in diminishing the strong intra-molecular attraction between FG chains and improving the formation of hydrogen bonds between GE and FG molecules [14]. Thus, it reduces the TS and increases the EB of composites by subsequently weakening the hydrogen bonds between FG chains. Such disruption and reconstruction of FG molecular chains decreases the rigidity and improves the flexibility of films by endowing them with more chain mobility. Similar results have been reported by Muscat et al.: they found that the elongation of polymeric materials depends on the mobility of their molecular chains [42].

As for the HTs addition on the tensile properties of composites, it is clear that the TS of films was greatly influenced by the addition of HTs. The TS of films was improved continuously by increasing HTs loadings for the FG/GE composites (*p* < 0.05). The improved TS of composites can be explained by the fact that firstly, there is a lot of intermolecular hydrogen bonding between HTs and FG, FG and GE, and GE and HTs, which could form a 3D network (see Scheme 1); and secondly, the large aspect ratio of the HTs is also beneficial to stress transfer [33].

The EB value increased with increasing content of HTs for the FG/GE composites with GE loadings from 0% to 30%. For FG/GE composites with GE loadings below 30%, EB values are low (<10%), indicating that these composites are still brittle even though plasticized by GE. After incorporation of HTs into FG/GE composites, HTs would act as a bridge to connect the fracture of films at the process of stressing, thus the EB of films was increased. However, when the GE loading was increased to 40%, the EB value reached 20%, which showed flexibility at a certain extent. When incorporating HTs into FG/GE 40%, the hydrogen bonding between HTs, FG, FG and GE hinders the sliding of the FG chains and consequently the EB values were decreased with the increase of HTs.

### 3.6. Moisture Uptake and Water Solubility Of Composites

**Figure 8:**
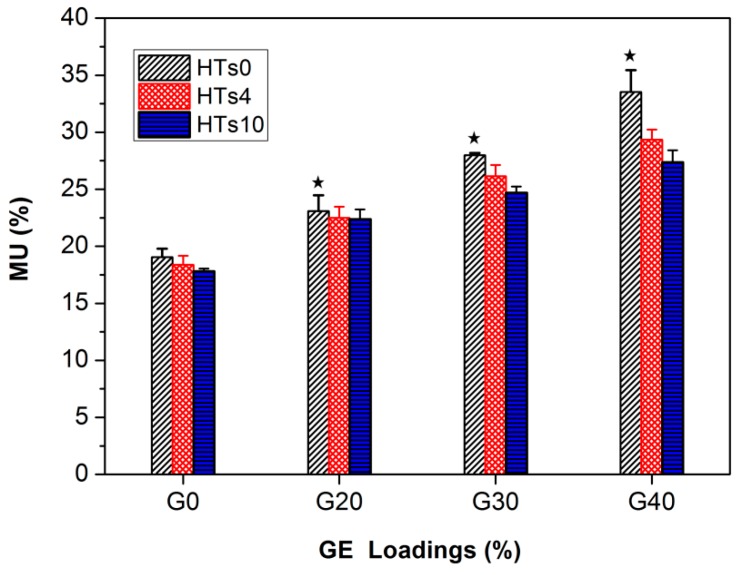
Moisture uptake of FG/GE/HT composites with various HT and GE loadings. * denotes significant difference between FG films containing different percentages of GE (*p* < 0.05).

Moisture uptake and water solubility are common parameters used to characterize the water-resistance of packaging materials [20]. The results for moisture uptake and water solubility of composite films are presented in Figure 8. The moisture uptake of composite films increased continuously with the increase of GE content (*p* < 0.05). This is due to the fact that the GE molecule has high hydroxyl density and is more hydrophilic than FG molecules. However, moisture uptake shows a decreased trend with the addition of HTs for all the FG/GE composites. This reduction in moisture uptake can be ascribed to the following reasons: Firstly, the formation of strong interactions via hydrogen bonds between FG, GE and HTs promotes the cohesiveness of the FG matrix and decrease its water sensitivity [43]. Secondly, moisture uptake of HTs is lower than that of FG and GE, so the addition of HTs reduces the moisture uptake of composites [33].

**Figure 9:**
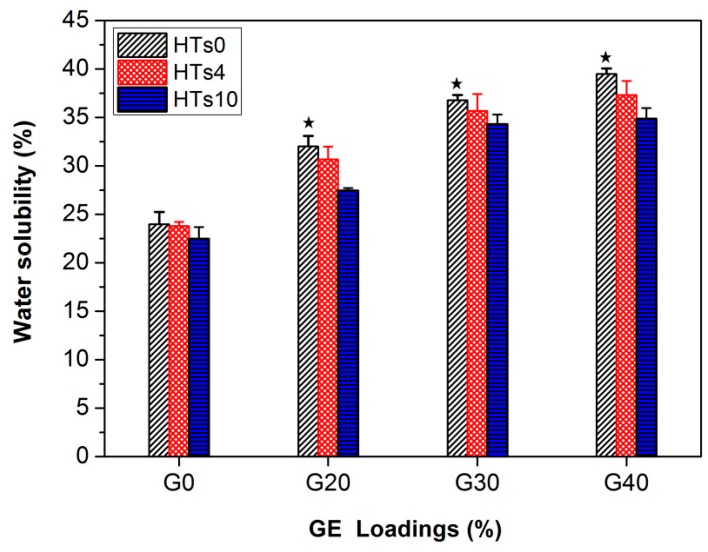
Water solubility of FG/GE/HT composites with various HT and GE loadings. * denotes significant difference between FG films containing different percentages of GE (*p* < 0.05).

The results of water solubility of composites are similar with those of moisture uptake, which also increased continuously with the increase of GE content (Figure 9) (*p* < 0.05). This behavior can be explained by the fact that GE has higher solubility than FG in the distilled water. In addition, it can be seen that water solubility decreased with the addition of HTs into the FG/GE composites. This is due to the presence of hydrogen bonds between FG, GE and HTs, and the insoluble property of HTs, which reduced the diffusion of water molecules in the composites and thus decreased the water solubility. Based on the above discussion, it was demonstrated that the incorporation of HTs could improve the water resistance properties of the composites.

**Figure 10:**
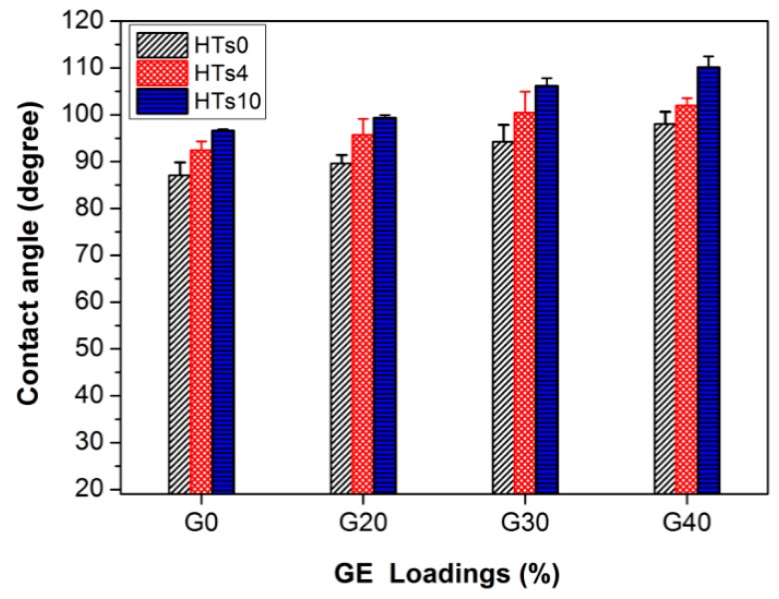
Contact angles of FG/GE/HT composites with various HT and GE loadings.

Another property of the films is their surface hydrophobicity, which was evaluated through WCA determination. The WCA of the pure FG and composites are shown in Figure 10. The WCA of pure FG film was approximately 87.07, and it increased with the increase of GE content in the composites. GE molecules can be inserted into the FG molecular chains, resulting in the formation of a more compact structure compared with a pure FG film. The compact structure of FG/GE composites blocked the diffusion of large amounts of water molecules into the FG matrix when the water was dropped onto the surface of the films. Thereby, the WCA of FG/GE composites increases with GE addition. When considering a certain GE loading, an increase in HTs content clearly brought an increase in WCA values of the FG/GE composites. This phenomenon can be attributed to the existence of hydrogen bonds among FG, GE and HTs, which also blocked the diffusion of large amounts of water molecules into the FG/GE films when the water was dropped onto the surface of composites.

### 3.7. Optical Properties

**Figure 11:**
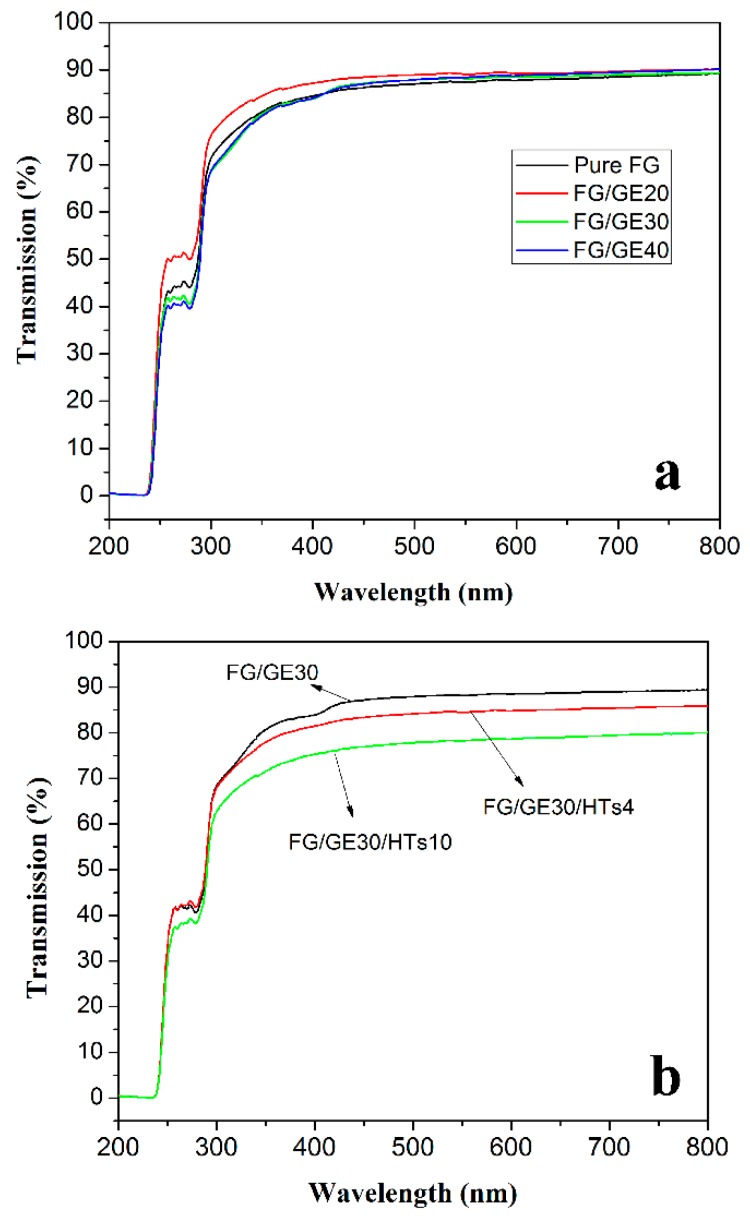
Optical properties of FG/HTs containing 0, 4 and 10 wt % (**a**) and FG/FG30/HTs containing 0, 4 and 10 wt % (**b**).

Transparency of packaging film is relevant to their functionality owing to their great impact on the appearance of the products. The light transmittance of the FG/GE films as a function of wavelength is presented in Figure 11a. It is observed that all the FG/GE composites films have high light transmission of above 80% in the visible region of 400–800 nm, which demonstrates that there is a great consistency between FG and GE, and the FG and GE reach molecule-level dispersion within the composites. Similar phenomena have been reported by researchers in kappa-carrageenan, plasticized with glycerol composite films [44].

On the other hand, light transmittance of FG/GE films showed a general decrease with increasing HTs loading (Figure 11b). Incorporation of HTs induces a decrease in the transparency of films based on FG/GE, owing to changes in light diffraction generated by the dispersed HTs. Film transparency decreases were dependent on the amount of HTs incorporated. It can be seen that the transmittance of FG/GE30 film decreased from 86% to 80% when 10% of HTs was added. Sothornvit et al. reported that transmittance of the PLA film decreased when HTs was added [45]. In spite of decreasing the transparency of films by the HTs, all films containing HTs showed above 75% transparency across the visible light regions (400–800 nm), indicating that the light transmittance of the resulting nanocomposites films was slightly affected by the HTs owing to their nanometer size and well-dispersed in the composites.

## 4. Conclusions

In summary, a series of FG-based composite films at different HTs loadings with and without GE as plasticizer, were prepared using the solution-casting method. Results indicated that the addition of GE greatly improves film flexibility with a decrease in the TS of film. Moisture uptake and water solubility were also improved by the addition of GE into the FG matrix, indicating that the water-resistance of film was decreased by the GE added. The addition of GE into FG matrix had no negative effect on the transparency of films. In addition, the incorporation of HTs into the FG/GE composites could improve the TS and water-resistance of films simultaneously, due to the formation of hydrogen-bonding networks in the composites. Moreover, the presence of GE enhances the dispersion of HTs in the FG matrix and thus enhanced the properties of obtained composite films.
